# Effect of the Toll-Like Receptor 4 Antagonist Eritoran on Retinochoroidal Inflammatory Damage in a Rat Model of Endotoxin-Induced Inflammation

**DOI:** 10.1155/2014/643525

**Published:** 2014-06-17

**Authors:** Feyzahan Ekici, Emine Esra Karaca, Şafak Korkmaz, Osman Yüksel, Özlem Gülbahar, Murat Alper, Sevim Ercan, Meral Or

**Affiliations:** ^1^Department of Ophthalmology, Recep Tayyip Erdogan University Medical School, 53020 Rize, Turkey; ^2^Department of Ophthalmology, Sorgun State Hospital, 66700 Yozgat, Turkey; ^3^Department of Ophthalmology, Duzce State Hospital, 81100 Duzce, Turkey; ^4^Department of General Surgery, Gazi University Medical School, 06560 Ankara, Turkey; ^5^Department of Medical Biochemistry, Gazi University Medical School, 06560 Ankara, Turkey; ^6^Department of Pathology, Dışkapı Training and Research Hospital, 06330 Ankara, Turkey; ^7^Department of Medical Pharmacology, Gazi University Medical School, 06560 Ankara, Turkey; ^8^Department of Ophthalmology, Gazi University Medical School, 06560 Ankara, Turkey

## Abstract

*Purpose*. We investigated the effect of eritoran, a Toll-like receptor 4 antagonist, on retinochoroidal inflammatory damage in an endotoxin-induced inflammatory rat model. *Methods*. Endotoxin-induced inflammatory model was obtained by intraperitoneal injection of 1.5 mg/kg lipopolysaccharide (LPS). Group 1 had control rats; in groups 2-3 LPS and 0.5 mg/kg sterile saline were injected; and in groups 4-5 LPS and 0.5 mg/kg eritoran were injected. Blood samples were taken and eyes were enucleated after 12 hours (h) (groups 2 and 4) or 24 hours (Groups 3 and 5). Tumor necrosis factor-*α* (TNF-*α*) and malondialdehyde (MDA) levels in the serum and retinochoroidal tissue and nuclear factor kappa-B (NF*κ*B) levels in retinochoroidal tissue were determined. Histopathological examination was performed and retinochoroidal changes were scored. *Results*. Eritoran treatment resulted in lower levels of TNF-*α*, MDA, and NF*κ*B after 12 h which became significant after 24 h. Serum TNF-*α* and retinochoroidal tissue NF*κ*B levels were similar to control animals at the 24th h of the study. Eritoran significantly reversed histopathological damage after 24 h. *Conclusions*. Eritoran treatment resulted in less inflammatory damage in terms of serum and retinochoroidal tissue parameters.

## 1. Introduction

Toll-like receptors (TLRs) are a family of pattern recognition receptors that recognize distinct molecular patterns associated with microbial pathogens [1]. TLR4 binding is required for the recognition of Gram-negative LPS. Once the TLR4 signaling pathway is triggered, inactive cytosolic NF*κ*B is activated and induces the synthesis and release of proinflammatory mediators, including TNF-*α*, cytokines, chemokines, adhesion molecules, reactive oxygen species, and reactive nitrogen radicals [2, 3]. During this inflammatory response, MDA is produced as a result of lipid peroxidation [4]. MDA is a degradation product of free oxygen radicals and arachidonic acid pathway metabolites [5] and was suggested as a marker of oxidative stress [6].

Recent studies have demonstrated the expression and the function of TLRs in the eye, with significant implications for a better understanding of ocular immunity and the pathogenesis of inflammatory eye diseases affecting the cornea, uvea, and retina. TLR4 is expressed on the corneal epithelium [7], corneal stromal fibroblasts [8], ciliary body, iris endothelial cells [9], uveal resident antigen presenting cells [10], and retina pigment epithelium [11].

Endotoxin-induced uveitis (EIU) is an important ocular inflammatory model [12]. Initially, EIU was described as an anterior uveitis model affecting the anterior chamber, iris, and ciliary body [12, 13]. Subsequently, retinal and choroidal involvement were demonstrated [14, 15]. Inflammatory changes such as endothelial cell damage, blood-retinal barrier (BRB) dysfunction, adhesion/migration/infiltration of polymorphonuclear leukocytes, protein leakage from vessel walls, and retinal cell damage were described in the retina and retinochoroidal microvascular system in EIU models [16]. When an inflammatory response is initiated using LPS to trigger the TLR pathway, proinflammatory mediators, including TNF-*α*, interleukins, adhesion molecules, and chemokines, are released. TNF-*α*, in particular, modulates BRB damage in the EIU model, which is related to polymorphonuclear leukocyte adhesion, endothelial cell apoptosis, and microvascular damage [17]. Eritoran tetrasodium (E5564) is suggested for sepsis therapy, belonging to a new class of drugs which inhibits LPS-induced inflammation by blocking TLR4 [3].

We investigated the changes in serum and tissue levels of TNF-*α*, MDA, and NF*κ*B and the histopathological scores in a rat model of ocular inflammation induced by systemic LPS administration and determined the improvement of these ocular inflammation parameters induced by eritoran, a TLR4 antagonist.

## 2. Materials and Methods

### 2.1. Study Design

This was a prospective randomized controlled experimental animal study. Wistar albino male rats weighing 240–310 g and at the age of 8 weeks were used. The animals were housed under a 12 h light-dark cycle and were fed on a standard diet until the experiment. The criteria of the Association for Research in Vision and Ophthalmology (ARVO) resolution on the use of animals were applied and the study was approved by the Gazi University Ethic Committee for Experimental Animals.

### 2.2. Study Procedures

Endotoxin-induced inflammatory model was obtained by the intraperitoneal injection of 1.5 mg/kg LPS (Sigma-Aldrich, Munich, Germany,* Escherichia coli*, serotype O111:B4). Eritoran (E5564, Eisai, Inc.) was used as a TLR4 antagonist. After establishing anesthesia with intramuscular 100 mg/kg ketamine (Ketalar, Eczacıbaşı, Istanbul, Turkey), blood samples were obtained from the vena cava. Eritoran and saline injections routed through tail vein concomitantly. Enucleation was performed after peritomy and the animals were sacrificed. The right eyes were used for the biochemical parameters and the left eyes were kept for the histopathological evaluation.

### 2.3. Study Groups

Fifty rats were investigated in five groups (*n* = 10). To assign the rats to the individual study groups, the Random Allocation Software program (Version 1.0.0^©^ Mahmood Saghaei, Isfahan University of Medical Sciences, Isfahan, Iran) was used. The study groups were as follows. Group 1 has control rats, no intervention; in group 2, 1.5 mg/kg LPS and 0.5 mg/kg (11 *μ*L) saline were injected, blood samples were taken, and enucleation was performed after 12 h; in group 3, 1.5 mg/kg LPS and 0.5 mg/kg saline were injected, blood samples were taken, and enucleation was performed after 24 h; in group 4, 1.5 mg/kg LPS and 0.5 mg/kg eritoran were injected, blood samples were taken, and enucleation was performed after 12 h; in group 5, 1.5 mg/kg LPS and 0.5 mg/kg eritoran were injected, blood samples were taken, and enucleation was performed after 24 h. Inclusion of the 12 h and 24 h groups allowed us to monitor the changes in the inflammation parameters within time in the serum and the retinochoroidal tissue as well as the change in eritoran efficacy.

### 2.4. Serum and Tissue Cytokine Levels

The levels of TNF-*α* (pg/mL) and MDA (U/mL) in the serum and the retinochoroidal tissue were assessed with commercially available enzyme-linked immunosorbent assay (ELISA) kits: Rat TNF-*α* ELISA (Sigma-Aldrich, Munich, Germany) and lipid peroxidation (MDA) ELISA (Cell Biolabs, Inc., San Diego, CA) according to the manufacturer's instructions. Retinochoroidal tissue was rinsed with 1X phosphate buffer solution (PBS) to remove excess blood, homogenized in 20 mL of 1X PBS, and stored overnight at ≤−20°C. After two freeze-thaw cycles to get rid of the cell membranes, the homogenates were centrifuged for 5 minutes at 5000 ×g. The MDA and TNF-*α* assays were performed using the OxiSelect MDA and TNF-*α* adduct ELISA kit. Tissue samples were diluted to 10 mg/mL in 1X PBS and 100 *μ*L from each sample was added in duplicate to the 96-well protein binding plate. Then, 200 mL of assay diluent was added per well and incubated for 1 h at room temperature (RT) on an orbital shaker. The plate was washed 3 times with 1X wash buffer before adding the diluted anti-MDA and anti-TNF-*α* antibody and incubated for 1 h at RT on the orbital shaker. Finally, after warming, the substrate solution was added to each well and the plate was incubated at RT. The absorbance of each sample was measured at 450 nm after the addition of stop solution using MultiSkan Go spectrophotometer (Thermo Scientific, Finland). The concentration of MDA (U/mL) and TNF-*α* (ng/mg protein) in the samples is then determined by comparing the optical density of the samples to the standard curve.

For the measurement of NF*κ*B (ng/mg protein) levels, retinochoroidal tissues from each group were centrifuged at 4°C, 1000 g for 5 minutes. The supernatant fluid was discarded and the cells were washed by PBS twice. The tissues were incubated in the complete lysis buffer for 30 min. Then lysis buffer was centrifuged at 4°C, 10.000 g for 10 min, and the supernatants were collected. Protein concentration was measured by using bicinchoninic acid method. Aliquots of each sample containing lg protein were fractionated on 10% polyacrylamide-sodium dodecyl sulfate gel (70 V at first, when the protein ran out of concentration gel, turned to 90 V for 1.5 h) and transferred to polyvinylidene fluoride (350 mA, 90 min) membrane. The membrane was blocked with 10% nonfat milk in PBS containing 0.9% saline and 0.05% Tween 200 (PBST) for 1 h at room temperature and then incubated with primary antibodies (NF*κ*B antibody diluted at 1 : 200, Santa Cruz, USA; b-actin antibody diluted at 1 : 1000, Santa Cruz, USA) at 4°C overnight. After washing for 5 min and 3 times with PBST, the blots were incubated with horse radish peroxidase conjugated anti-rabbit IgG second antibody (1 : 1000) for 1 h at RT. Immunoreactive band was visualized with enhanced chemiluminescence staining. The intensity of each band was scanned by software. The ratio of NF*κ*B/b-actin was considered as the relative expressional level of NF*κ*B.

### 2.5. Histopathological Analysis

The enucleated eye specimens were fixed in 10% formalin for 72 h and embedded in paraffin, and 5 mm tissue sections were taken through posterior pole and stained with hematoxylin-eosin staining for light microscopy. Histopathological findings assessed in the retina were scored as follows:* minimal* (few infiltrating cells, pigment disturbance, or minimal degeneration),* mild* (infiltration of anterior retina and thickening of the inner limiting membrane),* moderate* (retinal detachment and infiltration, some retinal folds),* severe* (retinal degeneration), and* very severe* (no retinal tissue observable) [18].

### 2.6. Statistical Analysis

The sample size required for this study and the power were calculated using the G∗Power program (Version 3.0.10, Franz FAUL, Kiel University, Germany, http://www.gpower.hhu.de/). To obtain 80% power, with effect extent *f* = 0.30, *α* = 0.05 type I error, and *β* = 0.20 type II error ratio, it was calculated that a minimum of 10 rats per group was needed.

The Shapiro-Wilk test indicated that only the serum TNF-*α* and tissue MDA levels after 12 h and 24 h showed a normal distribution. Results were presented as the minimum and the maximum values, the means ± standard deviation (SD), or the medians (interquartile range, [IQR]). To compare the levels of serum TNF-*α* and tissue MDA after 12 h and 24 h, one-way analysis of variance (ANOVA) and the Bonferroni test for post hoc pair comparison were used. For other comparisons, the Kruskal-Wallis nonparametric variance analysis was used. The variance among groups and pairwise comparisons were determined using the Mann-Whitney *U* test with Bonferroni correction. A paired-sample* t*-test for serum TNF-*α* and tissue MDA and a Wilcoxon signed rank test for the other parameters were used to evaluate the change between 12 h and 24 h for the same treatment. Histopathological scores were evaluated by Chi-square likelihood ratio. All statistical analyses and calculations were made with the MS Excel 2003 and SPSS programs (Statistical Package for Social Sciences version 15.0, SPSS Inc., Chicago, Illinois, USA). A statistical level of significance was defined as *P* ≤ 0.05.

## 3. Results

**Figure 1:**
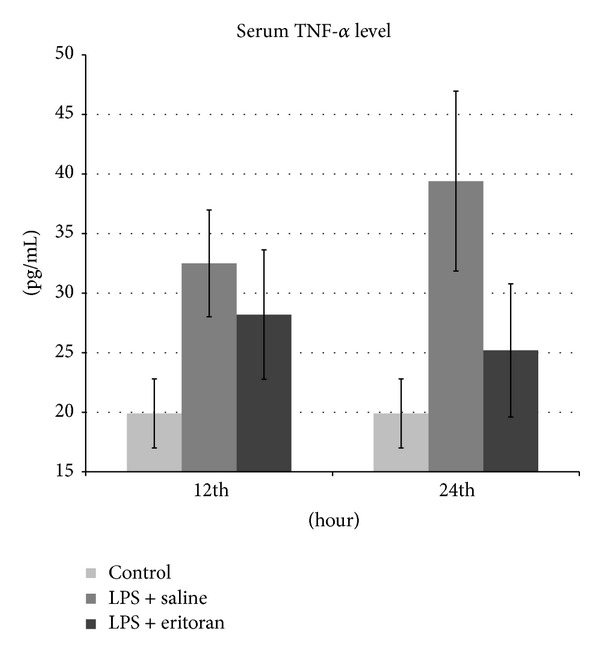
The tumor necrosis factor-*α* (TNF-*α*) levels in serum after 12 h and 24 h in control group with no intervention, rats with sepsis but without eritoran, and rats with sepsis treated with eritoran. Vertical lines show standard deviation. LPS: lipopolysaccharide.

**Figure 2:**
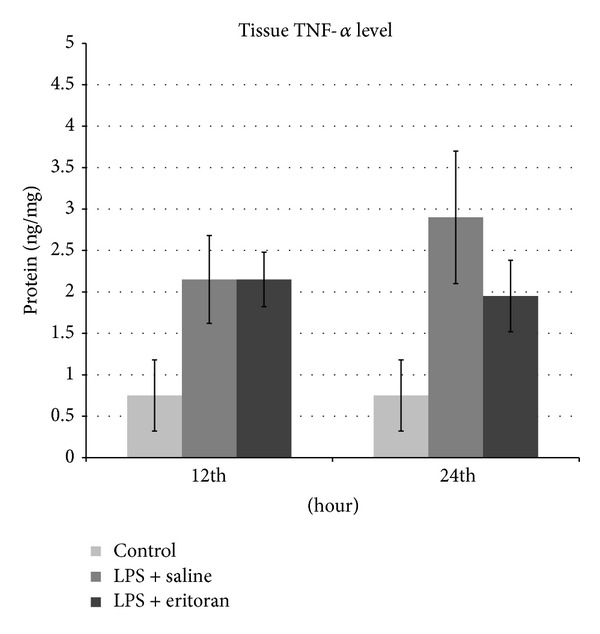
The tumor necrosis factor-*α* (TNF-*α*) levels in retinochoroidal tissue after 12 h and 24 h in control group with no intervention, rats with sepsis but without eritoran, and rats with sepsis treated with eritoran. Vertical lines show standard deviation. LPS: lipopolysaccharide.

**Figure 3:**
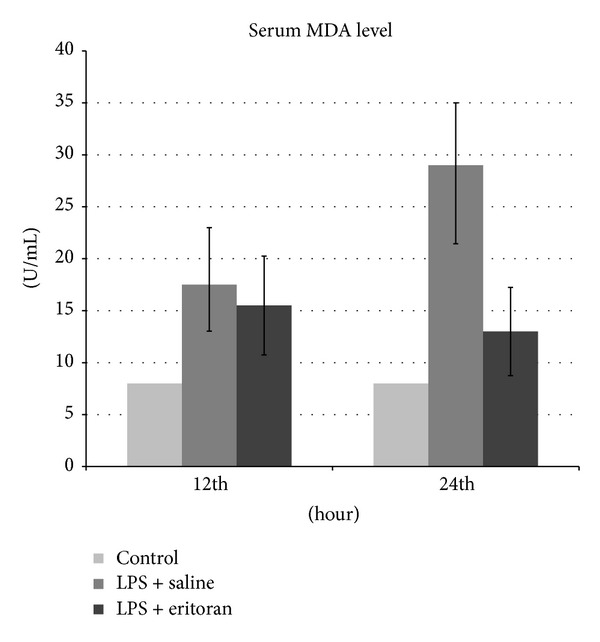
The malondialdehyde (MDA) levels in serum after 12 h and 24 h in control group with no intervention, rats with sepsis but without eritoran, and rats with sepsis treated with eritoran. Vertical lines show standard deviation. LPS: lipopolysaccharide.

**Figure 4:**
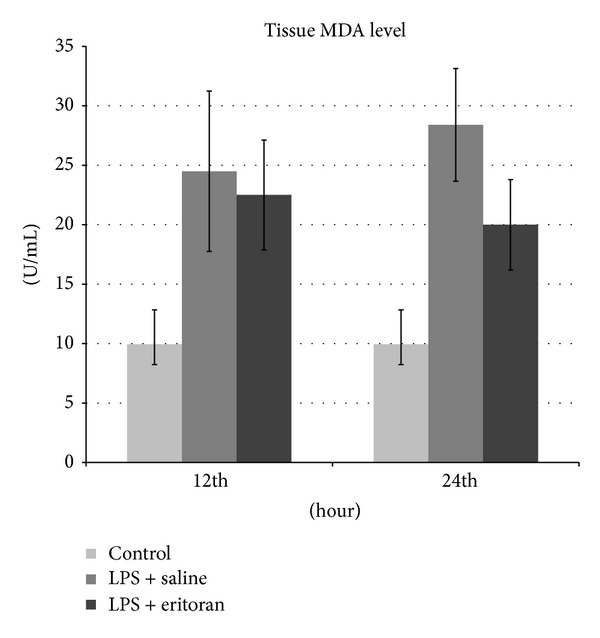
The malondialdehyde (MDA) levels in retinochoroidal tissue after 12 h and 24 h in control group with no intervention, rats with sepsis but without eritoran, and rats with sepsis treated with eritoran. Vertical lines show standard deviation. LPS: lipopolysaccharide.

**Figure 5:**
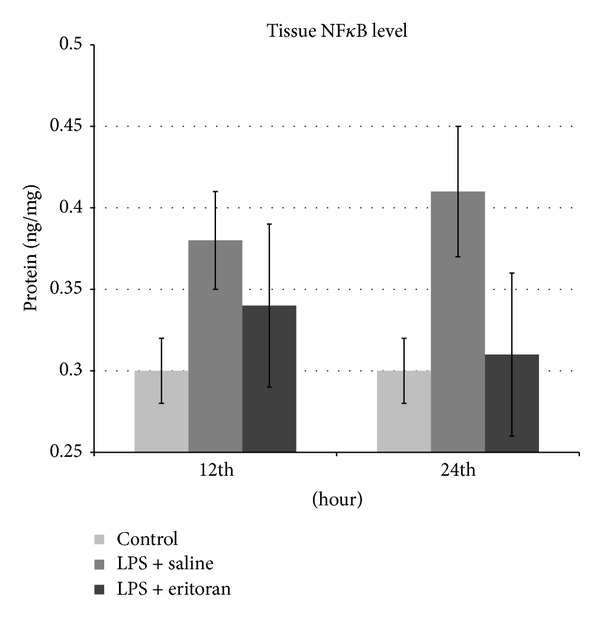
The nuclear factor kappa B (Nf*κ*B) levels in retinochoroidal tissue after 12 h and 24 h in control group with no intervention, rats with sepsis but without eritoran, and rats with sepsis treated with eritoran. Vertical lines show standard deviation. LPS: lipopolysaccharide.

The TNF-*α* and MDA levels in the serum and retinochoroidal tissue and the NF*κ*B level in the retinochoroidal tissue in control rats with no intervention (group 1), rats not treated with eritoran (groups 2 and 3), and rats treated with eritoran (groups 4 and 5) are shown in Table 1. The levels of these markers are also schematized in Figures 1–5 for the 12 h (groups 2 and 4) and 24 h (groups 3 and 5) measurements.

### 3.1. Serum and Tissue TNF-*α* Levels

The serum TNF-*α* levels were significantly higher in groups 2, 3, and 4 compared to the control group (*P* < 0.001), which confirmed that inflammation was present in groups 2, 3, and 4. The serum TNF-*α* level was significantly different between groups 3 and 5 (*P* < 0.001). Although the serum TNF-*α* level was higher in group 2 than group 4, this was not statistically significant (*P* = 0.112). Similarly, serum TNF-*α* levels were not significantly different between groups 1 and 5 (*P* = 0.139), indicating that treatment with eritoran caused similar serum TNF-*α* levels with control rats after 24 h (Table 1, Figure 1).

The tissue TNF-*α* levels in all groups were significantly different than the control group (group 1) (*P* < 0.001) and were also different between groups 3 and 5 (*P* < 0.001), but no significant difference was found between groups 2 and 4 (*P* = 0.353). These tissue TNF-*α* levels confirmed the inflammatory status of the animals (Table 1, Figure 2).

### 3.2. Serum and Tissue MDA Levels

Serum and tissue MDA levels were significantly different in all study groups (groups 2–5) compared to the control group (group 1) (*P* < 0.001) and between groups 3 and 5 (*P* < 0.001), but no significant difference was found between groups 2 and 4 (*P* = 0.105 for serum, *P* = 0.334 for tissue) (Table 1, Figures 3 and 4).

### 3.3. Tissue NF*κ*B Levels

Tissue NF*κ*B levels were significantly different in both 12 h groups (groups 2 and 4) and group 3 compared to the control group (group 1) (*P* < 0.001). The difference between groups 2 and 4 was not statistically significant (*P* = 0.218). Tissue NF*κ*B levels were not significantly different between groups 1 and 5 (*P* = 0.529), indicating that treatment with eritoran induced as lower tissue NF*κ*B levels as the control rats after 24 h (Table 1, Figure 5).

### 3.4. Comparison of 12- and 24-Hour Levels of TNF-*α*, MDA, and NF*κ*B

Serum and tissue TNF-*α* levels were significantly higher at 24th h of the inflammatory process (group 3) when compared to 12th h (group 2) (*P* = 0.008 and *P* = 0.004, resp.). In contrast, treatment with eritoran induced nonsignificant lower serum and tissue TNF-*α* levels after 24 h (group 5) compared to those measured after 12 h (group 4) (*P* > 0.05) (Table 1).

Serum and tissue MDA levels after 24 h of inflammation in group 3 were significantly higher when compared to group 2 (*P* = 0.004 and *P* = 0.003, resp.). In contrast, serum and tissue MDA levels were significantly lower with eritoran treatment after 24 h in group 5 compared to those measured after 12 h in group 4 (*P* = 0.007 and *P* = 0.002, resp.) (Table 1).

NF*κ*B levels in tissue were higher after 24 h in group 3 than after 12 h in group 2, although this difference was not significant (*P* = 0.073). On treatment with eritoran, the NF*κ*B levels were significantly lower after 24 h in group 5 compared to 12 h in group 4 (*P* = 0.006) (Table 1).

### 3.5. Histopathological Analysis

**Figure 6:**
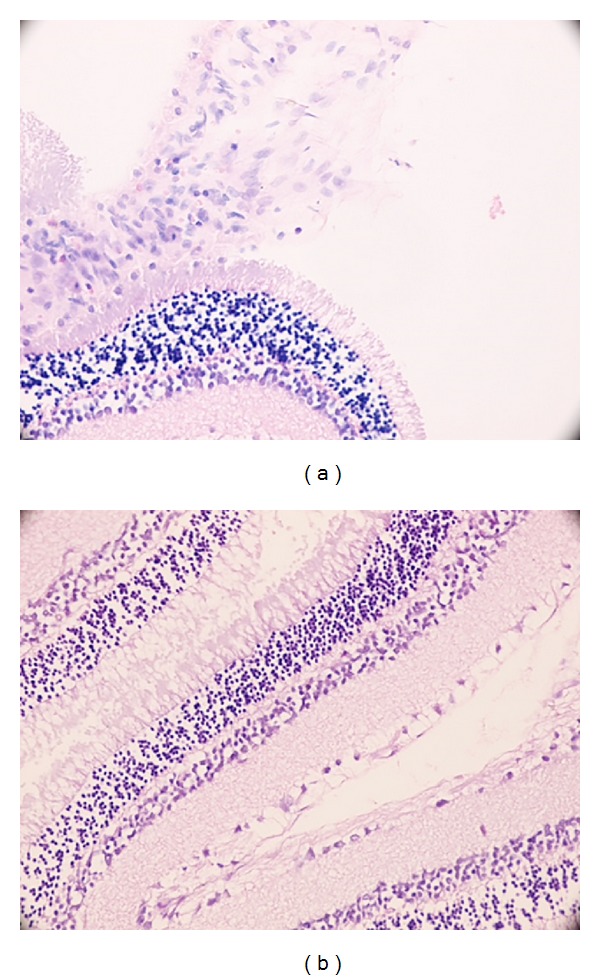
In histopathological section (H&E, ×400) of an eye from (a) one of group 3 rats (saline injected) at 24th h, see moderate retinal infiltration and some retinal folds and from (b) one of group 5 rats (eritoran injected) at 24th h, see minimal infiltrating cells.

The retinochoroidal sections were examined and the severity of inflammatory changes was scored as described before. The results of the scores were shown in Table 2. For statistical purposes, “minimal” results were labeled as “not affected” and mild or worse results were labeled as “affected.” Histopathological scores at 12 h were not significantly different between saline and eritoran groups. At 24 h of study LPS-induced inflammation related damage became more prominent in LPS + saline group. Also at 24th h, all saline injected rats were “affected”; however, 60% of eritoran treated rats remained “not affected” and this difference was statistically significant (*χ* ^2^ = 10.974; *P* = 0.001) (Table 3) (Figure 6).

## 4. Discussion

We investigated the effect of systemic LPS injection on retinochoroidal inflammation by assessing TNF-*α*, MDA, and NF*κ*B levels in serum and tissue and the changes in the histopathological scores. We further studied the effect of eritoran on retinochoroidal inflammation in this LPS-induced inflammation model. We found an improvement in all parameters upon treatment with eritoran. Therefore, we believe eritoran might be effective in preventing retinochoroidal inflammatory damage.

Eritoran is a TLR4 antagonist that binds to the MD-2 region, thus inhibiting LPSTLR4 binding and consequently NF*κ*B activation [19]. In vitro studies indicate that eritoran, without endotoxin-like activity upon binding to the TLR4 molecule [20], inhibits cytokine production in human myeloid and macrophage cultures [21] and downregulates intracellular cytokine production [22]. Recent studies using EIU models showed that the iris endothelium and ciliary body express TLR4, and TLRs play an important role in the pathogenesis of anterior uveitis [23]. Li et al. showed that in the iris-ciliary body complex expression of NF*κ*B was increased in a rat model of EIU [24]. TLR4 mutant rats did not respond to LPS and were protected from the lethal effect [25].

Eritoran prevents the uncontrolled inflammatory response and inhibits the proinflammatory mediator production during inflammatory process. TLR4 molecule is expressed in retinochoroidal tissue and may play a potential role in the ocular inflammatory processes. So, the main aim of this study with regard to TLR4 antagonism was whether retinochoroidal damage could be prevented with eritoran.

In this study, we observed significant high TNF-*α* levels in both serum and retinochoroidal tissue after the induction of inflammation with LPS. Serum and tissue TNF-*α* levels were significantly lower in eritoran injected animals; even serum TNF-*α* levels were the same as the control rats after 24 h. TNF-*α* is an important mediator of sepsis and is responsible for acute phase reactions, endothelial activation, capillary permeability changes, end organ damage, and shock-like syndrome [26]. Therefore, TNF-*α* is a target molecule for the treatment of sepsis and ocular inflammatory disease processes [27]. Evereklioglu et al. reported that TNF-*α* levels correlated with disease activity in Behcet's disease [28]. TNF-*α* expression was observed in the retina after 4 h of LPS injection and peaked at 22–24 h [29]. Because TNF-*α* plays an important role in the ocular inflammatory disease process, recent studies have reported successful results of anti-TNF-*α* therapies [30, 31]. Although there is no published report of changes in TNF-*α* levels in retinochoroidal tissue with TLR4 antagonism in the literature, we believe that through TLR4 inhibition the TNF-*α* load could be diminished in the retina and thus further tissue injury could be ameliorated.

MDA is a metabolite of free oxygen radicals and some inflammatory mediators, including thromboxane A2, and may be used as a marker of oxidative stress [4, 5]. MDA forms covalent bonds with intracellular proteins and DNA and also forms advanced toxic peroxidation end products [6]. Studies have shown that lipid peroxidation is responsible for retinal cell damage in experimental autoimmune uveitis models [32, 33]. Evereklioglu et al. reported that MDA levels correlated with disease activity in Behcet's disease [28]. Retinal damage could be prevented using antioxidant molecules against these peroxidation products [34, 35].

Similar to TNF-*α*, serum MDA levels were significantly higher in group 2 rats when compared to controls and continued to be higher in group 3. On treatment with eritoran, serum MDA levels were lower than saline injected groups at 12th h in group 4 rats and were significantly lower after 24 h in group 5 rats. However, in contrast to TNF-*α*, these levels did not return to those of the control rats after 12 h or 24 h. MDA is a metabolite of various proinflammatory molecules and thus the MDA load may be greater than that of TNF-*α*; hence, the serum MDA levels were not as low as the TNF-*α* levels when treated with eritoran. In addition, MDA forms covalent bonds with proteins; thus, this may be a cause for higher levels of MDA than that of TNF-*α*. We achieved a significant lower serum and tissue MDA levels with eritoran, suggesting that lipid peroxidation related retinochoroidal damage may be prevented by TLR4 antagonism.

NF*κ*B stimulates proinflammatory genes when the TLR signaling pathway is triggered and constitutes the most important step of the inflammatory process [36]. NF*κ*B correlates with the mortality rates in sepsis patients [37, 38], and with retinal damage in ocular inflammatory diseases, while NF*κ*B inhibition prevented further tissue damage in experimental models of autoimmune uveitis [39, 40].

Similar to serum TNF-*α* levels, NF*κ*B levels after 12 h in group 4 were nonsignificantly lower than saline injected group (group 2) and significantly lower at 24 h in group 5. Additionally, group 5 levels were nonsignificantly different than those of control rats after 24 h. This indicates that NF*κ*B production and further cascade of the inflammatory response could be inhibited by TLR4 antagonism. Although we clearly showed that NF*κ*B inhibited the excessive inflammatory response in a LPS-induced inflammation model, further studies are required to evaluate the immune response when an infectious agent exists.

In the saline injected groups (groups 2 and 3), inflammatory response worsened by time, and serum and retinochoroidal tissue TNF-*α* and MDA levels were significantly higher at 24 h when compared to 12 h. NF*κ*B levels were already high starting from the early stages of inflammation; thus no significant difference between 12 h and 24 h was identified. In the eritoran treated groups, although inflammation parameters were worse after 24 h than after 12 h, group 5 showed a better response to eritoran than group 4. This may be explained by the BRB effect. Eritoran is a large molecule (1400 kDa) and its capability to pass through the BRB is still unknown. We hypothesized that when inflammation worsened by time, disruption of the BRB might be increased, and thus the passage of eritoran may be more likely. Similarly, the histopathological evaluation suggested that systemically administered LPS caused retinochoroidal damage especially more severe at 24 h and some of this damage could be reversed by eritoran. As we compared 12 h and 24 h study groups, eritoran caused a nonsignificant reversal of inflammatory damage at 12 h but this trend was more prominent and significant at 24 h although sepsis parameters in the tissue have worsened. This explains at least in part why inflammatory parameters have worsened after 24 h, while at this time the passage of the drug would be easier and so a better response would be expected. As a limitation of our study, since the local behaviour of eritoran in the retinochoroidal tissue and BRB is unknown, we probably demonstrated the potential systemic beneficial effects of eritoran rather than a local effect. Further studies are needed to investigate the local effect of eritoran when administered intravitreally and the passage of eritoran through the BRB.

The change in the TNF-*α* level in the eritoran treated groups at 12 h (group 4) and 24 h (group 5) was not significant, whereas the changes in MDA levels were significant. Although the mechanism is unclear, this indicates that TNF-*α* levels were lowered more rapidly than that of MDA. As indicated above, this may be associated with a greater MDA load in tissues or covalent bonds between MDA and intracellular proteins.

This present study has several limitations. The effect of eritoran was tested only after one injection at 12th and 24th h of the study. The effect of eritoran on retinochoroidal damage should be investigated for a longer time period and with multiple injections as well.

In conclusion, both serum and retinochoroidal tissue levels of TNF-*α* and MDA and tissue levels of NF*κ*B improved in a rat model of LPS-induced inflammation when treated with eritoran, a TLR4 antagonist. To the best of our knowledge, this is the first report of the effect of eritoran on retinochoroidal inflammatory damage. Our data suggests that investigation of the effect of eritoran in other ocular diseases in which inflammatory processes may play a role including retinochoroiditis, posterior uveitis, senile macular degeneration, and diabetic retinopathy is warranted. Further investigations should evaluate the passage of eritoran through biological barriers, the toxic doses, and the results upon intravitreal administration.

## Figures and Tables

**Table 1:** TNF-*α*, MDA, and NF*κ*B levels of study groups.

Variable	Group 1 (control)	Group 2 (12 h) (LPS + saline)	Group 3 (24 h) (LPS + saline)	*P* value (group 2 versus 3)	Group 4 (12 h) (LPS + eritoran)	Group 5 (24 h) (LPS + eritoran)	*P* value (group 4 versus 5)	*P* value (group 1 versus 5)	*P* value (group 2 versus 4)	*P* value (group 3 versus 5)
Serum TNF-*α*	19.90 ± 2.89	32.50 ± 4.48	39.40 ± 7.55	0.008^a^	28.20 ± 5.43	25.20 ± 5.59	0.069^a^	0.139^c^	0.112^c^	<0.001^c^
	(15.00–24.00)	(25.00–39.00)	(22.0–48.00)		(19.00–36.00)	(15.00–34.00)				

Tissue TNF-*α*	0.75, 0.43	2.15, 0.53	2.90, 0.80	0.004^b^	2.15, 0.33	1.95, 0.43	0.057^b^	<0.001^c^	0.353^c^	<0.001^c^
	(0.30–1.00)	(1.90–2.70)	(2.20–3.60)		(1.70–2.40)	(1.40–2.90)				

Serum MDA	8.00, 8.00	17.50, 5.50	29.00, 6.00	0.004^b^	15.50, 4.75	13.00, 4.25	0.007^b^	<0.001^c^	0.105^c^	<0.001^c^
	(8.00-8.00)	(14.00–25.00)	(24.00–35.00)		(11.00–20.00)	(8.00–18.00)				

Tissue MDA	9.94 ± 1.71	24.50 ± 6.75	28.40 ± 4.74	0.003^a^	22.50 ± 4.62	20.00 ± 3.80	0.002^a^	<0.001^c^	0.334^c^	<0.001^c^
	(7.70–12.10)	(20.00–34.00)	(21.00–37.00)		(16.00–30.00)	(15.00–27.00)				

Tissue NF*κ*B	0.30, 0.02	0.38, 0.03	0.41, 0.04	0.073^b^	0.34, 0.05	0.31, 0.05	0.006^b^	0.529^c^	0.218^c^	<0.001^c^
	(0.27–0.32)	(0.32–0.77)	(0.35–0.46)		(0.28–0.37)	(0.24–0.36)				

^a^Paired-sample *t*-test, ^b^Wilcoxon signed rank test, ^c^Mann-Whitney *U* test.

TNF-*α*: tumor necrosis factor-*α*, MDA: malondialdehyde, Nf*κ*B: nuclear factor kappa B.

Data are presented as mean ± standard deviation (min–max) or median, interquartile range (min–max).

**Table 2:** Histopathological scores of study groups.

	Histopathological scores		LPS + saline	LPS + eritoran	Total
12th hour	Minimal	*n* (%)	7 (70.0)	9 (90.0)	16 (80.0)
	Mild	*n* (%)	3 (30.0)	1 (10.0)	4 (20.0)
	Moderate	*n* (%)	0 (0.0)	0 (0.0)	0 (0.0)
	Severe	*n* (%)	0 (0.0)	0 (0.0)	0 (0.0)
	Very severe	*n* (%)	0 (0.0)	0 (0.0)	0 (0.0)
	Total	*n * ** (%)**	**10 (100.0)**	**10 (100.0)**	**20 (100.0)**

24th hour	Minimal	*n* (%)	0 (00.0)	6 (60.0)	6 (30.0)
	Mild	*n* (%)	9 (90.0)	4 (40.0)	13 (65.0)
	Moderate	*n* (%)	1 (10.0)	0 (0.0)	1 (5.0)
	Severe	*n* (%)	0 (0.0)	0 (0.0)	0 (0.0)
	Very severe	*n* (%)	0 (0.0)	0 (0.0)	0 (0.0)
	Total	*n * ** (%)**	**10 (100.0)**	**10 (100.0)**	**20 (100.0)**

LPS: lipopolysaccharide.

**Table 3:** Relabeled statistical analysis of histopathological scores.

	Histopathological score		LPS + saline	LPS + eritoran	Total	χ^2^	P value
12 h	Not affected	*n* (%)	7 (70.0)	9 (90.0)	16 (80.0)	1.297	0.255
	Affected	*n* (%)	3 (30.0)	1 (10.0)	4 (40.0)		

24 h	Not affected	*n* (%)	0 (0.0)	6 (60.0)	6 (30.0)	10.974	**0.001**
	Affected	*n* (%)	10 (100.0)	4 (40.0)	14 (70.0)		

LPS: lipopolysaccharide; *χ* ^2^: Chi-square likelihood ratio; bold: statistically significant.

## References

[1] Krawczyk-Michalak K, Glapiński A, Brzezińska-Błaszczyk E (2008). Toll-like receptors and their role in regulation of the inflammatory response in sepsis. *Anestezjologia Intensywna Terapia*.

[2] Wiersinga WJ, van der Poll T, Vincent JL (2006). The role of toll-like receptors in sepsis. *Yearbook of Intensive Care and Emergency Medicine*.

[3] Wittebole X, Castanares-Zapatero D, Laterre PF (2010). Toll-like receptor 4 modulation as a strategy to treat sepsis. *Mediators of Inflammation*.

[4] Marnett LJ (1999). Lipid peroxidation—DNA damage by malondialdehyde. *Mutation Research: Fundamental and Molecular Mechanisms of Mutagenesis*.

[5] Pryor WA, Stanley JP (1975). A suggested mechanism for the production of malonaldehyde during the autoxidation of polyunsaturated fatty acids. Nonenzymatic production of prostaglandin endoperoxides during autoxidation. *Journal of Organic Chemistry*.

[6] Del Rio D, Stewart AJ, Pellegrini N (2005). A review of recent studies on malondialdehyde as toxic molecule and biological marker of oxidative stress. *Nutrition, Metabolism and Cardiovascular Diseases*.

[7] Song PI, Abraham TA, Park Y (2001). The expression of functional LPS receptor proteins CD14 and toll-like receptor 4 in human corneal cells. *Investigative Ophthalmology and Visual Science*.

[8] Kumagai N, Fukuda K, Fujitsu Y, Lu Y, Chikamoto N, Nishida T (2005). Lipopolysaccharide-induced expression of intercellular adhesion molecule-1 and chemokines in cultured human corneal fibroblasts. *Investigative Ophthalmology and Visual Science*.

[9] Brito BE, Zamora DO, Bonnah RA, Pan Y, Planck SR, Rosenbaum JT (2004). Toll-like receptor 4 and CD14 expression in human ciliary body and TLR-4 in human iris endothelial cells. *Experimental Eye Research*.

[10] Chang JH, McCluskey P, Wakefield D (2004). Expression of toll-like receptor 4 and its associated lipopolysaccharide receptor complex by resident antigen-presenting cells in the human uvea. *Investigative Ophthalmology and Visual Science*.

[11] Kindzelskii AL, Elner VM, Elner SG, Yang D, Hughes BA, Petty HR (2004). Toll-like receptor 4 (TLR4) of retinal pigment epithelial cells participates in transmembrane signaling in response to photoreceptor outer segments. *Journal of General Physiology*.

[12] Rosenbaum JT, Tabarra KF, Cello RM (1984). Endotoxin-induced uveitis. *Animal Models of Ocular Diseases*.

[13] Rosenbaum JT, McDevitt HO, Guss RB, Egbert PR (1980). Endotoxin-induced uveitis in rats as a model for human disease. *Nature*.

[14] Yang P, de Vos AF, Kijlstra A (1996). Macrophages in the retina of normal Lewis rats and their dynamics after injection of lipopolysaccharide. *Investigative Ophthalmology and Visual Science*.

[15] Ruiz-Moreno JM, Thillaye B, de Kozak Y (1992). Retino-choroidal changes in endotoxin-induced uveitis in the rat. *Ophthalmic Research*.

[16] Bhattacherjee P, Williams RN, Eakins KE (1983). An evaluation of ocular inflammation following the injection of bacterial endotoxin into the rat foot pad. *Investigative Ophthalmology and Visual Science*.

[17] Koizumi K, Poulaki V, Doehmen S (2003). Contribution of TNF-*α* to leukocyte adhesion, vascular leakage, and apoptotic cell death in endotoxin-induced uveitis in vivo. *Investigative Ophthalmology and Visual Science*.

[18] Kalsow CM, Dwyer AE (1998). Retinal immunopathology in horses with uveitis. *Ocular Immunology and Inflammation*.

[19] Kim HM, Park BS, Kim J-I (2007). Crystal structure of the TLR4-MD-2 complex with bound endotoxin antagonist eritoran. *Cell*.

[20] Rossignol DP, Lynn M (2002). Antagonism of in vivo and ex vivo response to endotoxin by E5564, an synthetic lipid A analogue. *Journal of Endotoxin Research*.

[21] Mullarkey M, Rose JR, Bristol J (2003). Inhibition of endotoxin response by E5564, a novel toll-like receptor 4-directed endotoxin antagonist. *Journal of Pharmacology and Experimental Therapeutics*.

[22] Czeslick E, Struppert A, Simm A, Sablotzki A (2006). E5564 (Eritoran) inhibits lipopolysaccharide-induced cytokine production in human blood monocytes. *Inflammation Research*.

[23] Chen W, Hu X-F, Zhao L, Li S, Lu H (2010). Toll-like receptor 4 expression in macrophages in endotoxin-induced uveitis in Wistar rats. *Chinese Journal of Ophthalmology*.

[24] Li S, Lu H, Hu X, Chen W, Xu Y, Wang J (2010). Expression of TLR4-MyD88 and NF-B in the iris during endotoxin-induced uveitis. *Mediators of Inflammation*.

[25] Qureshi ST, Larivière L, Leveque G (1999). Endotoxin-tolerant mice have mutations in toll-like receptor 4 (Tlr4). *Journal of Experimental Medicine*.

[26] Movat HZ, Cybulsky MI, Colditz IG, Chan MK, Dinarello CA (1987). Acute inflammation in gram-negative infection: endotoxin, interleukin 1, tumor necrosis factor, and neutrophils. *Federation Proceedings*.

[27] van der Poll T, Lowry SF (1995). Tumor necrosis factor in sepsis: mediator of multiple organ failure or essential part of host defense?. *Shock*.

[28] Evereklioglu C, Er H, Türköz Y, Cekmen M (2002). Serum levels of TNF-alpha, sIL-2R, IL-6, and IL-8 are increased and associated with elevated lipid peroxidation in patients with Behçet's disease. *Mediators of Inflammation*.

[29] de Vos AF, Klaren VNA, Kijlstra A (1994). Expression of multiple cytokines and IL-1RA in the uvea and retina during endotoxin-induced uveitis in the rat. *Investigative Ophthalmology and Visual Science*.

[30] Pleyer U, Mackensen F, Winterhalter S, Stübiger N (2011). Anti-TNF-*α* treatment for uveitis. Analysis of the current situation. *Ophthalmologe*.

[31] Neri P, Zucchi M, Allegri P, Lettieri M, Mariotti C, Giovannini A (2011). Adalimumab (Humira*™*): a promising monoclonal anti-tumor necrosis factor alpha in ophthalmology. *International Ophthalmology*.

[32] Goto H, Wu G-S, Chen F, Kristeva M, Sevanian A, Rao NA (1992). Lipid peroxidation in experimental uveitis: sequential studies. *Current Eye Research*.

[33] Rao NA (1990). Role of oxygen free radicals in retinal damage associated with experimental uveitis. *Transactions of the American Ophthalmological Society*.

[34] Wu G-S, Walker J, Rao NA (1993). Effect of deferoxamine on retinal lipid peroxidation in experimental uveitis. *Investigative Ophthalmology and Visual Science*.

[35] Bosch-Morell F, Romá J, Marín N (2002). Role of oxygen and nitrogen species in experimental uveitis: anti-inflammatory activity of the synthetic antioxidant ebselen. *Free Radical Biology and Medicine*.

[36] Liu SF, Malik AB (2006). NF-kappa B activation as a pathological mechanism of septic shock and inflammation. *American Journal of Physiology: Lung Cellular and Molecular Physiology*.

[37] Böhrer H, Qiu F, Zimmermann T (1997). Role of NF*κ*B in the mortality of sepsis. *The Journal of Clinical Investigation*.

[38] Arnalich F, Garcia-Palomero E, López J (2000). Predictive value of nuclear factor *κ*B activity and plasma cytokine levels in patients with sepsis. *Infection and Immunity*.

[39] Kubota S, Kurihara T, Mochimar H (2009). Prevention of ocular inflammation in endotoxin-induced uveitis with resveratrol by inhibiting oxidative damage and nuclear factor-*κ*B activation. *Investigative Ophthalmology and Visual Science*.

[40] Kitamei H, Iwabuchi K, Namba K (2006). Amelioration of experimental autoimmune uveoretinitis (EAU) with an inhibitor of nuclear factor-*κ*B (NF-*κ*B), pyrrolidine dithiocarbamate. *Journal of Leukocyte Biology*.

